# Posterior ventral tegmental area-nucleus accumbens shell circuitry modulates response to novelty

**DOI:** 10.1371/journal.pone.0213088

**Published:** 2019-03-05

**Authors:** Hailong Li, Jessica M. Illenberger, Michael N. Cranston, Charles F. Mactutus, Kristen A. McLaurin, Steven B. Harrod, Rosemarie M. Booze

**Affiliations:** Department of Psychology, Program in Behavioral Neuroscience, University of South Carolina, Columbia, South Carolina, United States of America; University of Kentucky, UNITED STATES

## Abstract

Dopamine release in the nucleus accumbens from ventral tegmental area (VTA) efferent neurons is critical for orientation and response to novel stimuli in the environment. However, there are considerable differences between neuronal populations of the VTA and it is unclear which specific cell populations modulate behavioral responses to environmental novelty. A retroDREADDs (designer drugs exclusively activated by designer receptors) technique, comprising designer G protein-coupled receptors exclusively activated by designer drugs and modulated by retrograde transported Cre, was used to selectively stimulate neurons of the VTA which project to the nucleus accumbens shell (AcbSh). First, the selectivity and expression of the human M3 muscarinic receptor-based adeno-associated virus (AAV-hM3D) was confirmed in primary neuronal cell cultures. Second, AAV-CMV-GFP/Cre was infused into the AcbSh and AAV-hSyn-DIO-hM3D(Gq)-mCherry (a presynaptic enhancer in the presence of its cognate ligand clozapine-N-oxide) was infused into the VTA of ovariectomized female Fisher 344 rats to elicit hM3D(Gq)-mCherry production specifically in neurons of the VTA which synapse in the AcbSh. Finally, administration of clozapine-N-oxide significantly altered rodents’ response to novelty (e.g. absence of white background noise) by activation of hM3D(Gq) receptors, without altering gross locomotor activity or auditory processing *per se*. Confocal imaging confirmed production of mCherry in neurons of the posterior aspect of the VTA (pVTA) suggesting these neurons contribute to novelty responses. These results suggest the pVTA-AcbSh circuit is potentially altered in motivational disorders such as apathy, depression, and drug addiction. Targeting the pVTA-AcbSh circuit, therefore, may be an effective target for pharmacological management of such psychopathologies.

## Introduction

It is well established that the ventral tegmental area (VTA) is a heterogeneous structure in the midbrain [1]. Neurons within the VTA regulate processes contributing to an array of behaviors involving reward salience and motivated action by forming specialized neuronal populations that project to different regions such as the nucleus accumbens shell (AcbSh), medial prefrontal cortex, and amygdala [1–3]. Specifically, when dopaminergic communication between the VTA and AcbSh is blocked, rodents fail to display increases in locomotor activity that are associated with novelty in the environment [4]. Likewise, optogenetic stimulation of GABAergic neurons in the VTA disrupts reward consumption by inhibiting dopamine release to the Acb [5]. As such, disruption of the VTA-AcbSh circuit has been implicated in the etiology of depression, drug addiction, and apathy. However, a greater understanding of which VTA cell subpopulations contribute to specific reward-related behaviors, i.e. novelty, is necessary to resolve which mechanisms underlie such motivational disorders.

A contemporary chemogenetic approach, designer receptors exclusively activated by designer drugs (DREADDs), is receiving increasing use to introduce and modulate G protein-coupled receptors (GPCRs) within a region of interest [6–7]. The human M3 muscarinic receptor coupled to Gq (hM3D(Gq)) is the most frequently used designer receptor characteristic of enhancing neuronal activity [7] and is activated in the presence of its cognate ligand, clozapine-N-oxide (CNO) [8]. Adeno-associated virus (AAV) delivery systems are combined with the flip-excision (FLEX)-switch method to introduce expression of the selected designer receptor (i.e. hM3D(Gq)) exclusively in cells comprising a circuit of interest [9]. Briefly, the viral vector contains 2 pairs of heterotypic, oppositely oriented loxP sites cloned with an inverted coding sequence. Contained within the vector, hM3D(Gq) and mCherry are only reoriented for encoding after Cre recombinases are retrograde transported to the virus-containing region down axonal projections. Additionally, cell-type-specific promoters, such as hSyn are used to limit viral encoding to neurons within the targeted circuit of interest. While there has been some controversy over the effectiveness of peripherally-administered CNO to activate DREADDs, the current literature supports that intraperitoneal (i.p.) administration of CNO, used in the current experiment, can indeed increase electrophysiological activity (measured by local field potential, single-unit recordings [10], multi-unit recordings [11], and enhanced c-Fos expression [12]) selectively in animals with the designer hM3D(Gq) receptor present.

The present experiment combined the described AAV delivery system with retrograde labeling to identify populations of dopaminergic neurons in the VTA-AcbSh circuitry that contribute to the expression of novelty-induced locomotor activity in rodents. It was hypothesized that stimulation of hM3D(Gq) receptors on dopaminergic VTA cells would enhance rodents’ locomotor response to novelty in the environment without influencing general motor activity. In particular, we were interested in the role of the posterior VTA (pVTA) in novelty behaviors. As the recruitment of mesolimbic cells is likely influenced by biological sex, ovariectomized females were used in the current experiment to determine the influence pVTA cell activation has over novel behaviors in the absence of cycling hormones. Parsing out how cells of the mesolimbic pathway contribute to novelty-related behaviors is critical to understanding the etiology of motivational disruptions present in many psychopathologies.

## Materials and methods

### Viral construction

**Fig 1:**
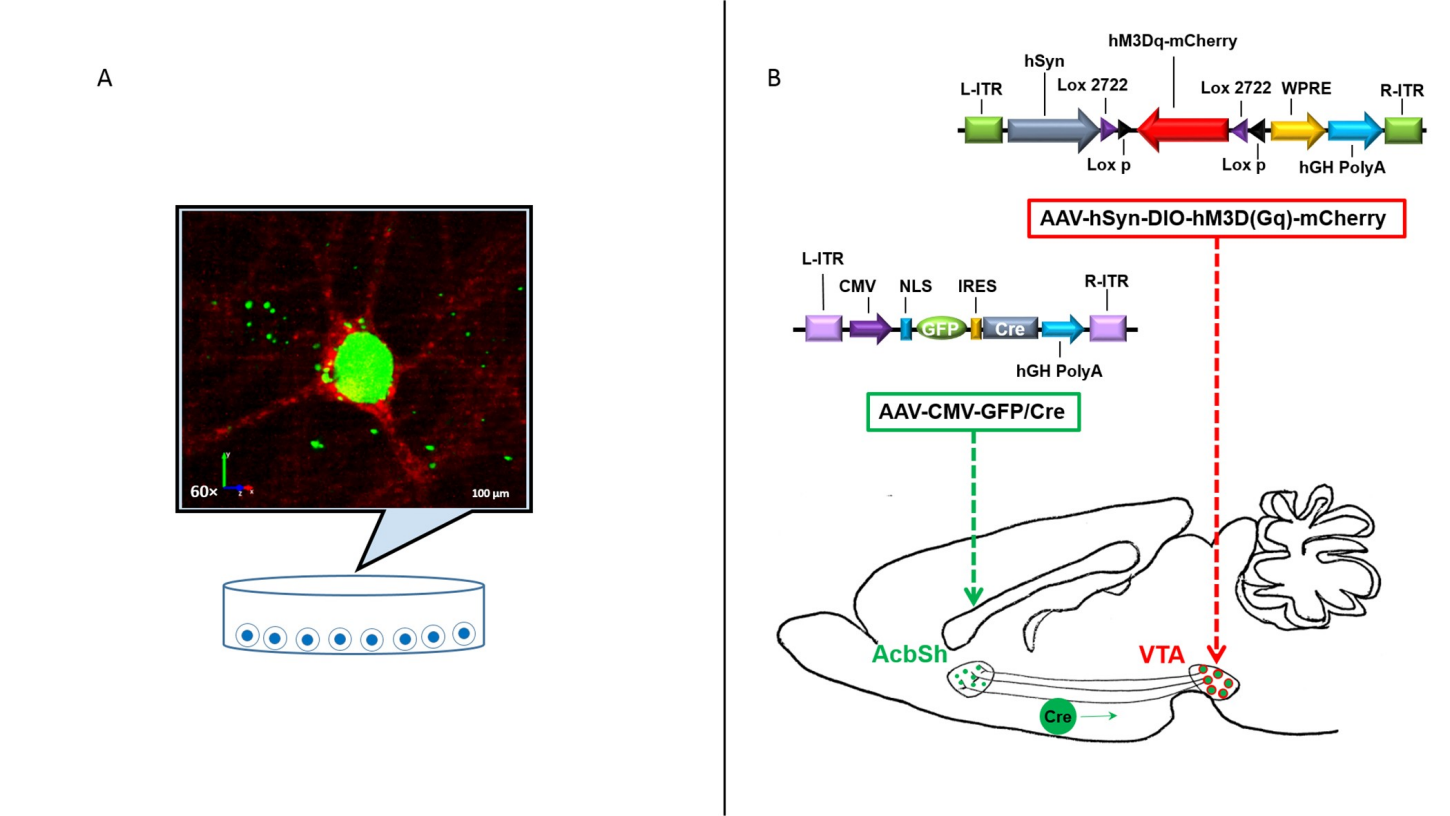
AAV FLEX-switch delivery system promotes selective Cre and hM3D(Gq) expression in primary cortical neurons. The current experiment utilized 2 viral vectors shown at the top of panel B. At the C-terminal of the AAV-CMV-GFP/Cre (serotype 9) virus, Cre was fused with green fluorescent protein (GFP) that can be observed in infected cells using a confocal microscope. The AAV-hSyn-DIO-hM3D(Gq)-mCherry virus contains a double inverted coding sequence promoted by the neuron-specific promoter, hSyn, and the mCherry red fluorescent marker that can similarly be observed in infected cells using a confocal microscope. A) A representative confocal image of GFP/Cre and hM3D(Gq)-mCherry expression in a primary cortical neuron is shown. Here, the GFP green fluorescence indicates the expression of Cre and is localized in the nuclei of cells. mCherry red fluorescence indicates the expression of hM3D(Gq) receptors after the vector was reoriented by GFP/Cre recombinase *in vitro*. 3-D reconstruction based on Z-stack scanning verified that mCherry fluorescence exhibited a membrane-specific distribution. B) When injected into the AcbSh of Fisher 344 rats, GFP/Cre is retrogradely transported along the axons of VTA neurons to the soma where it reorients hM3D(Gq)-mCherry as represented in the drawing.

As presented in Fig 1, the AAV-CMV-GFP/Cre (serotype 9) (addgene #49056) virus used in the current experiment contained a cDNA encoding Cre recombinase fused with green fluorescent protein (GFP) at the C-terminal. The AAV containing a double inverted coding sequence of hM3D(Gq) promoted by a neuron-specific promoter (hSyn) was generated and fused with mCherry red fluorescent marker at the C-terminal (AAV-hSyn-DIO-hM3D(Gq)-mCherry). The AAV-hSyn-DIO-hM3D(Gq)-mCherry viral vector was constructed at the Viral Vector Core Facilities at the University of South Carolina.

### Primary neuronal cell culture

Expression of mCherry was first verified in primary *in vitro* cell cultures to ensure proper reorientation of the AAV containing hM3D(Gq) by Cre from the AAV-CMV-GFP/Cre (serotype 9) virus. Cortical regions were dissected for *in vitro* culture on gestational day 18 from Fisher 344 rat fetuses (Harlan Laboratories, Indianapolis, IN, USA) as described previously [13]. After dissection, cortical tissue was incubated in a solution of 2 mg/ml trypsin in Hank’s balanced salt solution (HBSS) buffered with 10 mM HEPES (GIBCO Life Technologies, Grand Island, NY, USA) for 10 min, and then washed with HBSS following trypsin inhibitor treatment. Next, cells were distributed to 12 well glass-bottom dishes (MatTek Corporation, Ashland, MA, USA) coated with poly-L-lysine and cultured at 37°C in a 5% CO_2_/95% room air-humidified incubator. Fresh Neurobasal medium was supplemented at weekly intervals. Primary cortical neurons (cultured for 7 days after dissection) were infected with AAV-CMV-GFP/Cre (serotype 9) and AAV-hSyn-DIO-hM3D(Gq)-mCherry for 7 days before imaging by confocal microscope.

Z-stack images were obtained with a Nikon TE-2000E confocal microscope utilizing Nikon's EZ-C1 software (version 3.81b) for the analysis of hM3D(Gq) expression in primary cell cultures and later in tissues obtained after behavioral testing. Two-dimensional virtual scanning was performed with a Nikon Eclipse E800 fluorescence microscope with MBF Bioscience’s Stereo Investigator software.

### Animal subjects and stereotaxic surgeries

Thirty two adult ovariectomized female Fisher 344/N rats (Harlan Laboratories, Indianapolis, IN) were pair-housed in a controlled environment under a 12-hour light/12-hour dark cycle (lights on at 7:00h/ lights off at 19:00h) with *ad libitum* access to 20/20X chow (Harlan Teklad, Madison, WI) and water. Each animal underwent stereotaxic surgery, in which the animal was anesthetized with sevoflurane (Abbot Laboratories, North Chicago, IL: catalog #035189) and placed within the stereotaxic apparatus (Kopf Instruments, Tujunga, CA: Model 900) with the scalp exposed. Two small 0.40 mm diameter holes were drilled into the skull in neuroanatomical locations relative to Bregma in order to infuse the viral vectors (2 μl AAV-CMV-GFP/Cre serotype 9, (10^12^ vg/ml), or 1.5 μl AAV-hSyn-DIO-hM3D(Gq)-mCherry serotype 2 (10^12^ vg/ml)) into the AcbSh (0.5 mm lateral, 1.2mm rostral to Bregma, 7 mm depth) and VTA (1 mm lateral, 5 mm caudal to Bregma, 8 mm depth) respectively [14]. A 10 μl Hamilton syringe (catalog #1701) was used to infuse viral vectors at a rate of 0.2 μl/min. Twenty two animals received sham surgeries (i.e., no viral infusions) to serve as controls. All procedures were carried out in strict accordance with the recommendations in the Guide for the Care and Use of Laboratory Animals of the National Institutes of Health. The protocol for this research methodology was approved by the Institutional Animal Care and Use Committee at the University of South Carolina (animal assurance number: D16-00028).

### Locomotor testing

Following at least 14 days of recovery from surgery, animals received 3–5 days of habituation to the locomotor testing apparatus, before activity testing sessions began. Hamilton-Kinder locomotor activity monitors were square (40 cm × 40 cm) enclosures paired with Motor Monitor software (Hamilton Kinder Inc., Ponway, CA) that recorded the number of photocell (32 emitter/detector pairs) interruptions within each 5-min period of each 60-min behavioral testing session. A Plexiglas insert (~ 40 cm diameter) was placed into the chambers to transform the chambers into a round field design; the manufacturer tuned the photocell emitter/detector pairs to account for the width of the Plexiglas inserts. Behavioral testing occurred between 9:00 and 11:00 AM (EST), during the light phase of the day, under dim lighting conditions (< 10 lx). The dependent variable of interest was total ambulations (the sum of x and y photocell interruptions) in normal/no novelty conditions and then in novelty conditions following administration of saline or clozapine-N-oxide (Sigma-Aldrich, St. Louis, MO; catalog #c0832). During testing sessions with normal/no novelty conditions, white noise (70 dB) was present throughout 60-min sessions. To test each animal’s response to novelty without intruding on its ability to move freely about the cage, the white background noise was turned off for a 10-min period, 30 min after the start of testing sessions. The 10-min duration was chosen to ensure that at least one of the 5-min “bins” encapsulated the novel stimulus (silence) response while also presumably allowing for time after the novel stimulus is removed to observe activity levels return to normal.

### CNO dose response

The pattern of locomotor activity following variable doses of CNO was examined during 5 (1/day) sessions with normal conditions (i.e. no novelty present). A total of 13 animals, 9 that received a sham surgery (sham animal) and 4 that received viral infusions (DREADDs animals), received an i.p. injection of either saline or 0.15, 0.3, 0.6, or 1.0 mg/kg CNO immediately prior to 1-hour locomotor testing sessions. These doses of CNO were chosen based off evidence that similar doses (0.1 and 0.3 mg/kg) activated hM3D(Gq) to effectively increase locomotor behavior [10]. CNO doses were presented in a Latin-square design. The remaining 7 animals received saline once and only the 1 mg/kg dose of CNO prior to 4 sessions with no novelty present.

### Novelty

After establishing the general pattern of locomotor activity in response to CNO administration, 14 sham animals and 10 DREADDs animals were tested under conditions with novelty (i.e. 10-min absence of white noise) present in the environment. All animals received an i.p. saline injection immediately prior to 1 session and a 1 mg/kg CNO injection prior to 4 sessions in which novelty was present. The novelty response in the absence of hM3D(Gq) activation is described by the pattern of activity displayed during 2 sessions following saline administration (1 session with no novelty and 1 session with novelty). The influence of hM3D(Gq) receptor activation by CNO on the novelty response is defined by average patterns of activity displayed by sham or DREADDs animals across 4 novelty sessions following CNO administration.

### Gap-prepulse inhibition

Following a single session to allow animals to habituate to the testing chamber, the auditory processing ability of each animal was tested using gap prepulse inhibition (gap-PPI) in a reflex modification paradigm based on the mammalian acoustic startle reflex [15] immediately following saline or CNO injection. The startle platform (SR-Lab Startle Reflex System, San Diego Instruments, Inc., San Diego, CA) is enclosed in a 10 cm-thick double-walled, 81 × 81 × 116 cm dark acoustic isolation cabinet (external dimensions) (Industrial Acoustic Company, INC., Bronx, NY), instead of the 1.9 cm-thick ABS plastic or laminate cabinets offered with this system. Deflection of the test cylinder generated by animals’ responses to the auditory startle-evoking pulse was converted into analog signals by a piezoelectric accelerometer integral to the bottom of the cylinder. Each session began with a 5-min acclimation period under continuous 70 dB(A) white background noise followed by 6 pulse-only auditory startle response (ASR) trials with a fixed 10 msec intertrial interval (ITI). Six testing trial blocks consisted of the presentation of a 20-msec gap in the white background noise at an interstimulus interval (ISI) of 0, 30, 50, 100, 200, or 4000 msec prior to the auditory startle stimulus (100 dB(A), 20-msec duration). The absence of white noise during gap-PPI sessions served as a conditioned cue to alert the animal that an auditory startle pulse was imminent. During a single session, each ISI condition was presented 6 times (1/trial block) in a Latin Square design. ISIs of 0 and 4000 msec were included to provide a measure of baseline ASR. For all testing trials, the ITI was variable (15–25 msec range).

### Histology

Immediately following behavioral testing, animals were sacrificed and brains were extracted to confirm expected GFP/Cre and hM3D(Gq)-mCherry expression in cells of the AcbSh and VTA respectively. All animals were deeply anesthetized with sevoflurane and transcardially perfused with ~100 ml of 100 mM PBS followed directly by ~150 ml of chilled 4% paraformaldehyde (PFA) buffered in PBS. Brains were post-fixed in 4% chilled PFA overnight then sectioned in 50 μm-thick coronal slices using a vibratome. Sequential slices were then placed in a 24-well plate with 1 ml PBS until image processing.

### Statistical analyses

Mixed-factorial analysis of variance (ANOVA) techniques were used to examine alterations to locomotor activity and gap-PPI (SPSS Statistics 25, IBM Corp., Somers, NY). First, to determine if CNO dose-dependently altered locomotor activity under normal/no novelty conditions, a dose (5; saline, 0.15, 0.3, 0.6, and 1.0 mg/kg CNO) × time (12; 1 hour divided into 5 min “bins”) repeated-measures ANOVA was conducted on data from animals exposed to all 5 dose conditions (n = 13). Second, to establish the pattern of activity under novel conditions and to ensure baseline novelty responses were not different between sham and DREADDs animals, a time (7; period following onset of novel stimulus divided into 5 min “bins”) × novelty (2) × surgery (2; DREADDs: n = 10, Sham: n = 2) mixed-factor ANOVA was conducted on data collected from sessions following saline injections. Additional regression analyses (Graphpad V5.02, GraphPad Software, Inc., La Jolla, CA) were employed to illustrate the markedly different response functions for the two conditions. Third, to determine if stimulating hM3D(Gq)-expressing cells of the VTA modified the response to novelty, data collected from sessions following 1 mg/kg CNO injections were analyzed in a time (7) × surgery (2; DREADDs: n = 10, Sham: n = 14) mixed-factor ANOVA. Orthogonal decompositions were used to determine if activity across time or interactions involving time followed a linear, quadratic, or cubic trend. Again, additional regression analyses were employed to illustrate the markedly different response functions for the two surgery conditions. Fourth and finally, the peak ASR amplitude values, collected in gap-PPI, were analyzed to assess the animals’ auditory processing ability. An injection (2; saline and CNO) × surgery (2) × ISI (6; 0, 30, 50, 100, 200, 4000) mixed-factor ANOVA was conducted. Orthogonal decompositions were used to determine if auditory processing across ISI or interactions involving ISI followed a linear, quadratic, or cubic trend. Effect sizes (η_p_^2^), which have a maximum value of 1.0, are presented for all statistically significant findings.

## Results

### Validation of hM3D(Gq) and Cre expression using in vitro cortical neurons

The representative fluorescently labeled cell shown in Fig 1A confirms the efficiency of AAV expression: GFP green fluorescence was primarily localized in the nuclei of neurons infected with AAV-CMV-GFP/Cre and mCherry red fluorescence signal was clearly shown in primary cortical neurons. Moreover, scanning through Z-stacked images of mCherry expression within neurons showed a clear membrane-specific distribution of hM3D(Gq). Together, these results confirm that the double inverted coding sequence of hM3D(Gq)-mCherry was successfully reoriented by Cre in infected primary cortical neurons. The expression pattern of the displayed cell is representative of what was expected in cells of the VTA infused with the AAV-hSyn-DIO-hM3D(Gq)-mCherry virus and receiving GFP/Cre via retrograde transport from the AcbSh.

### Locomotor activity with Novelty and hM3D(Gq) activation

**Fig 2:**
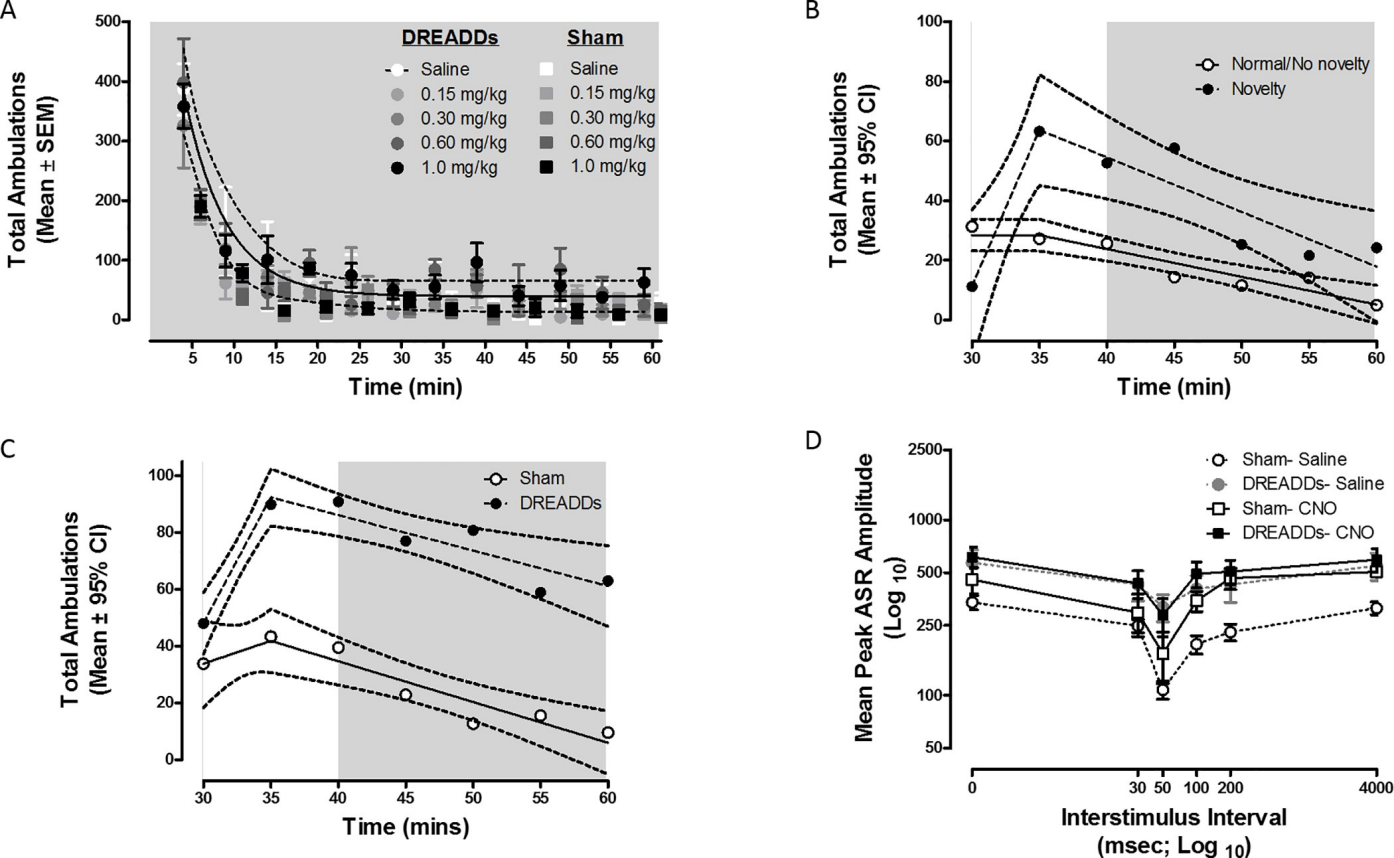
Locomotor response to CNO under normal conditions. **A)** Four animals that had received viral infusions and nine that received sham surgeries were exposed to various doses of CNO (0, 0.15, 0.3, 0.6, or 1.0 mg/kg) across 5 days under normal/no novelty present conditions. The mean number of total ambulations (± SEM) did not significantly change as a function of CNO dose, indicating that the administered doses of CNO did not elicit unforeseen locomotor effects. **B)** 12 animals (2 sham and 10 DREADDs) were administered 1 ml/kg 0.9% saline prior to a 60 min normal/no novelty present testing session and a novelty present testing session. The absence and presence of white background noise is indicated by the white and grey scale backgrounds, respectively. Only activity recorded after the 30-min time point (when white noise was temporarily turned off during the novelty present condition) was analyzed and is presented here. The mean number of total ambulations generally decreased over 30–60 min. However, in the presence of novelty, animals increased activity and sustained high activity for approximately 15–20 min. **C)** Twenty-two animals (10 DREADDs and 12 sham) were administered 1 mg/kg CNO prior to four novelty present testing sessions. The mean number of total ambulations increased after white noise was removed from the environment but gradually decreased once the novelty stimulus was removed. Animals that received viral infusions rather than sham surgeries increased activity in response to novelty to a greater degree and sustained high levels of activity for a longer period of time, suggesting that stimulation of hM3D(Gq) receptors on neurons of the VTA-AcbSh circuitry prolongs animals’ locomotor response to novelty. **D)** Following the conclusion of locomotor testing, all animals were administered 1 mg/kg saline or 1 mg/kg CNO prior to gap-PPI testing sessions to ensure that no animals exhibited abnormal auditory processing. Mean peak ASR amplitude (±SEM) across 2 days is displayed, with maximal inhibition observed at the 50 msec ISI. Neither viral infusions nor CNO administration significantly disrupted inhibition of the ASR, as indicated by statistically similar ASR amplitude curves.

Recent experiments have suggested that CNO, the ligand used to stimulate the hM3D(Gq) receptor, or its metabolite clozapine, can alter behavior distinct from the effects attributed to designer receptor activation [16]. To determine if CNO significantly influenced locomotor activity in a dose-dependent manner, four animals that had received viral infusions were exposed to saline and various doses of CNO (0.15 mg/kg, 0.3 mg/kg, 0.6 mg/kg, and 1.0 mg/kg) in a Latin-square design prior to 1-hour activity testing sessions under normal/no novelty conditions. CNO was not expected to significantly alter behavior during these testing sessions, first, because the targeted circuit is not involved in gross motor activity (in contrast to the substantia nigra), and second, CNO is pharmacologically inert outside of its effects on hM3D(Gq) receptors in rats. A dose × time repeated-measures ANOVA did not support a significant change in total ambulations as a function of CNO dose (dose: F(4,48) < 1.0) as displayed in Fig 2A. Overall, animals decreased locomotor activity precipitously in the first 15 min of testing before activity stabilized to an asymptotic level (quadratic effect of time: F(1,12) = 111.6, p ≤ 0.001, η_p_^2^ = 0.9). Using GraphPad Prism 5 (version 5.02), a nonlinear (one-phase decay) regression was fit to the data and indicated that the rate at which activity decreased across time was not significantly different between doses (F(9,690) = 1.15, p ≤ 0.32) with the rate of the global fit at approximately 0.2936 ± 0.0523. Stimulation of the hM3D(Gq) receptor by CNO did not appear to significantly influence locomotor activity under normal/ no novelty conditions.

The pattern of locomotor activity following saline administration in the presence of a novel stimulus was established first using a novelty × time × surgery mixed-factor ANOVA. Prior to the novelty × time × surgery mixed-factor ANOVA, a novelty × surgery mixed-factor ANOVA verified that groups had statistically similar (F(1,10) < 1.0) average levels of activity leading up to the onset of the novel stimulus (i.e., up to the 30-min time point). Locomotor activity levels were expected to significantly increase following the onset of the novelty stimulus at the 30-min time point. Animals generally decreased activity over the 30–60 min period analyzed (linear effect of time: F(1,10) = 5.08, p ≤ 0.048, η_p_^2^ = 0.34). However, Fig 2B illustrates that animals sustained high levels of activity for a longer period of time in the presence of novelty compared to when novelty was not present (quadratic effect of novelty × time: F(1,10) = 5.3, p ≤ 0.04, η_p_^2^ = 0.35). The significant novelty X time interaction was further analyzed with segmental linear regressions fit to the datasets from novelty and normal/no novelty conditions, as shown in Fig 2B. Weighting the sums of squares by the inverse of the standard deviation squared resulted in R square values of 0.9 and 0.93 for the normal/no novelty and novelty fits, respectively. Rather than a global fit, the analysis indicated that different parameters were needed to appropriately characterize the data from each condition (F(3,8) = 15.6, p ≤ 0.001). The intercept and slope to the inflection point (X_0_ = 35) were significantly different between the two fits (intercept: F(1,8) = 22.03, p ≤ 0.002; slope1: F(1,8) = 23.51, p ≤ 0.001). Animals in the novelty condition increased activity when white noise was turned off, resulting in a positive slope (9.58 ± 1.64) of activity in the first five minutes analyzed. Animals in the normal/no novelty condition did not increase activity, resulting in a slope (-1.27 ± 1.27) that was not significantly different from zero (F(1,4) < 1.0) in the first five minutes analyzed. These results emphasize the difference between the two conditions by characterizing activation of the novelty response by a positive slope in activity following removal of the white noise.

Next, a time × surgery mixed-factor ANOVA was conducted on average (4 session) locomotor activity following the onset of a novel stimulus to determine how stimulating hM3D(Gq) receptors influenced the locomotor response to novelty. Prior to the time × surgery mixed-factor ANOVA, a univariate ANOVA with surgery as the between-subjects factor, verified that groups had statistically similar (F(1,20) = 2.47 p ≤ 0.13) average levels of activity leading up to the onset of the novel stimulus (i.e., up to the 30-min time point). Animals generally increased activity after white noise was removed from the environment, reaching peak ambulations around 35 minutes into the session (sham: 43.36 ± 13.24; DREADDs: 89.9 ± 15.67). Once novelty was removed (i.e. background noise was turned back on at the 40-min time point) activity gradually decreased (quadratic effect of time: F(1,22) = 9.99, p ≤ 0.005, η_p_^2^ = 0.31). Animals that received viral infusions rather than sham surgeries, however, increased activity to a greater degree and sustained higher levels of activity for a longer period of time after the white noise was turned back on (quadratic effect of time × surgery: F(1,22) = 6.25, p ≤ 0.02, η_p_^2^ = 0.22). The time X surgery interaction was further analyzed by segmental linear regressions fit to the datasets from sham and DREADDs animals, as shown in Fig 2C. Weighting the sums of squares by the inverse of the standard deviation squared resulted in R square values of 0.81 and 0.94 for sham and DREADDs groups, respectively. The analysis indicated that different parameters were needed to appropriately characterize the data from each surgery condition (F(3,8) = 20.4, p ≤ 0.001). More specifically, the intercepts and ascending slopes representing activity between the 30 and 35 minute time points were significantly different (intercepts: F(1,8) = 5.08, p ≤ 0.05; slope1: F(1,8) = 6.54, p ≤ 0.03) with DREADDs animals displaying a greater increase in activity following the offset of white noise (sham: 0.26 ± 2.13; DREADDs: 8.9 ± 1.15). These results suggest that following CNO administration, animals which received viral infusions had a greater initial response to novelty than sham animals. Together, the results support the hypothesis that activation of hM3D(Gq) receptors, expected following CNO administration, on neurons of the VTA alters the locomotor response to novelty, without altering general locomotor activity.

### Gap-prepulse inhibition

To ensure that neither stereotaxic surgeries nor CNO injections impaired animals’ auditory processing system, all rats were tested for gap-PPI of the ASR twice each in the absence and presence of CNO. Robust inhibition to the gap in background noise, illustrated in Fig 2D, was observed independent of surgery (i.e., viral infusions vs. sham surgeries) or CNO administration suggesting the integrity of auditory information processing. Overall, peak inhibition (ASR: 227.36 ± 71.87) was displayed when the 20-msec prepulse gap in background white noise occurred approximately 50 msec prior to the startle auditory stimulus. A injection × surgery × ISI mixed-factor ANOVA confirmed these observations. Statistically similar ASR amplitude curves suggest no alterations in auditory information processing in animals that received viral infusions compared to sham surgeries (surgery × ISI: F(5,50) < 1.0) nor in animals that received CNO compared to saline prior to testing (injection × ISI: F(5,50) < 1.0). Additionally, CNO administration did not disrupt inhibition of the ASR in either sham or DREADDs animals (surgery × injection × ISI: F(5,50) < 1.0). Thus, the results support the integrity of the animals’ auditory processing system.

### Validation of hM3D expression in VTA neurons

**Fig 3:**
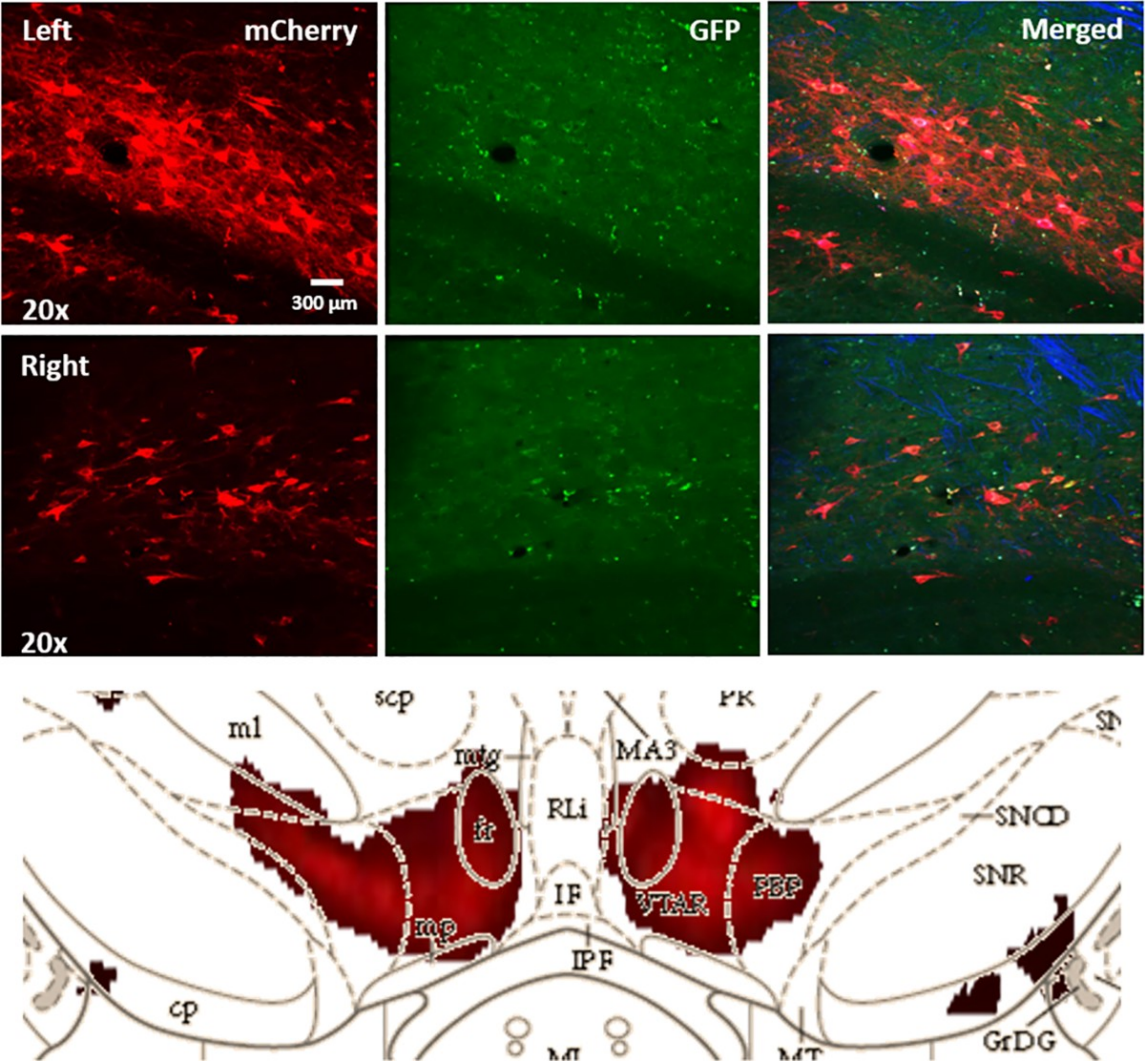
Histological identification of DREADDs expression. Virus-infused cortical tissues were sectioned into 50μm thick coronal slices to determine which neuronal VTA subpopulations were infected by the AAV-hSyn-DIO-hM3D(Gq)-mCherry virus and stimulated by CNO. hM3D(Gq) receptors (as indicated by mCherry expression) were predominantly expressed in neurons of the pVTA. Blue DAPI (4’,6-diamidino-2-phenylindole) staining suggest that hM3D(Gq)-mCherry expression was limited to a subset of pVTA neurons that communicate with the AcbSh.

Finally, to confirm the expression of hM3D(Gq)-mCherry in the VTA, all rats were perfused and their brains were sectioned into 50 μm-thick coronal slices using a Pelco easiSlicer vibratome (Ted Pella Inc., Redding, CA). In Fig 3, the images show that the fluorescent red mCherry signals of hM3D(Gq) were primarily expressed in neurons of the pVTA. Two-dimensional-virtual tissue scan with MBF Bioscience’s Stereo Investigator software further confirmed the distribution of hM3D(Gq) in the pVTA of rat brains.

## Discussion

The VTA-AcbSh circuit is recognized for eliciting responses to natural rewards. Pathophysiological impairment of the projections among this circuit are critical to emotional and motivational disorders, such as drug addiction and depression [1, 5, 17]. However, it is unclear which neuronal subpopulations within the heterogeneous VTA contribute to responses to novelty in the environment. The objective of the current experiment was to investigate which components of the VTA-AcbSh circuitry modulate novelty responses. Combining the cell-type-specific AAV delivery system with retrograde labeling in *in vitro* cortical neurons lead to membrane-specific mCherry expression exclusively in cells that received GFP/Cre recombinase from retrograde transportation along axons of VTA projections to the AcbSh. *In vivo*, stimulating hM3D(Gq) receptors with i.p. administration of 1 mg/kg CNO extended the period of increased locomotor activity following the onset of a novel stimulus (e.g. the removal of white background noise) without significantly altering gross motor behavior or auditory processing. Confocal imaging of GFP and mCherry fluorescent signals in extracted brain tissues revealed that hM3D(Gq)-expressing cells were located primarily in the pVTA. These results add to previous evidence of anterior-posterior heterogeneity in the VTA [18] and identifies the novelty response as one of the behaviors which is mediated by pVTA-AcbSh circuitry.

A concern when employing DREADDs methods is whether the hM3D(Gq) receptor ligand CNO, utilized in many DREADDs-based experiments, is pharmacologically inert outside of the targeted response. A recent experiment by Manvich et al., 2018 [16] demonstrated that the CNO metabolite, clozapine, can produce behavioral effects in rats distinct from those effects produced by stimulation of hM3D(Gq) receptors. However, the 10 mg/kg dose of CNO shown to produce behavioral effects was at least 10X greater than the doses used in the current experiment (0.15, 0.3, 0.6, 1.0 mg/kg); none of which significantly altered locomotor activity when administered to rats under conditions with no novelty present. Consistent with the results of Manvich et al., 2018 [16], the 1.0 mg/kg dose of CNO failed to produce detectable levels of intravenous plasma clozapine 30 or 60 min after i.p. injection. Similarly, the 1 mg/kg dose of CNO did not alter PPI in ovariectomized females in the current experiment or male Long-Evans rats in an experiment by MacLauren et al., 2016 [19]. Unlike MacLauren et al. [19], the acoustic startle reflex was not significantly reduced by administration of 1 mg/kg CNO in the current experiment. The 1 mg/kg CNO dose was thus inferred to be capable of stimulating hM3D(Gq) receptors to alter responses to novelty, but incapable of eliciting significant off-target effects. Nevertheless, in the present studies, sham animals that had received the same 1 mg/kg CNO dose as DREADDs animals were used as a control group in the final analysis of locomotor behavior to ensure the influence of VTA-AcbSh circuitry stimulation in the presence of novelty was appropriately described.

Because CNO failed to alter the activity of sham animals, it is assumed that the observed effect of CNO on behavior is dependent upon the presence of the designer hM3D(Gq)-mCherry receptor. Further, because activity following saline was not significantly different between DREADDs and sham animals, the results suggest that CNO administration is also necessary for the prolonged novelty response observed. Thus, although not verified through *in vivo* electrophysiological methods [such as 10–12], changes to behavior following CNO administration compared to following saline suggest that cells expressing hM3D(Gq)-mCherry were stimulated with the administration of CNO. The results of this preliminary dose-response experiment suggest that CNO doses of 1 mg/kg is viable to use in experiments in which total ambulation is the dependent variable.

Novelty paradigms frequently mentioned within the current literature include those introducing a novel object to the animal’s environment or by placing the animal in a novel environment [20]. However, an object from a familiar environment can also be removed to create a novel environment for the animal [21]. The novelty paradigm used in the current experiment is different from commonly described paradigms (e.g. the standardized novel object recognition task) in that the novel stimulus is silence (i.e. the removal of background noise) rather than a physical object. Using silence as a novel stimulus provided the opportunity to observe progressive decreases in activity once the novel stimulus was removed. The current results add to the existing literature by demonstrating that the locomotor response to novelty is comparable when rodents’ recognize a change in the auditory (described here) and visual environment (as described in the current literature). More so, each animal’s ability to recognize silence as a change in environment was assessed in the gap-PPI paradigm. Given recognized visual acuity variation in rodents across different strains [22], it would benefit future researchers to similarly explore novelty responses via different modalities and to additionally test subjects’ acuity in said modality through a paradigm such as PPI ([23], see also [24]).

The pVTA, where neurons expressing hM3D(Gq)-mCherry were located in the present studies, is highly populated with dopaminergic neurons relative to the anterior VTA (aVTA) which possesses relatively more GABAergic neurons [25]. Functional differences along the anterior-posterior gradient of the VTA are possibly due to the preferential targeting of different subnuclei, different connectivity of various cell types, and also differential expression of membrane receptors and channels. However, the different afferents and efferents of the aVTA vs. pVTA also likely contribute to the functional differences [26]. The results of the present experiment support existing evidence that the AcbSh receives strong innervation from the pVTA but little innervation from the aVTA. In addition, the current experiment adds to previously described functions of the pVTA-AcbSh circuitry specifically by demonstrating its contribution to the novelty response in rodents.

While hSyn is a neuron-specific promoter, it is clear that a variety of pVTA cell types express (as seen in Fig 3) hM3D(Gq)-mCherry and were thus stimulated by CNO administration in the current experiment. Similar to evidence surrounding dopamine function, recent studies focusing on VTA GABA and glutamate functions reveal functional heterogeneity in the locomotor, rewarding, and reinforcing properties of drugs of abuse administered along the anterior-posterior gradient [25, 26]. For example, GABA receptor agonists increase locomotor activity when administered to the pVTA but not the aVTA [27]. Likewise, rats self-administer ethanol into the pVTA but not the aVTA, and self-administration of ethanol into the pVTA increases the locomotor activity [28]. Indeed, injection of ethanol into the pVTA increases extracellular dopamine levels in the ventral pallidum and in the medial prefrontal cortex, while administration into the aVTA fails to do so [29].

The primary aim of the current experiment was to identify specific cell populations of the pVTA-AcbSh which contribute to behavioral responses to novelty. The current literature suggests that general activity (the dependent measure of the current experiment) varies as a function of estrous cycle [30] and gonadal hormones can influence object recognition [31] and spatial memory [32, 33]. Thus, to ensure within-group and within-subject differences could not be attributed to cycling gonadal hormone levels, all of the animals used in the current experiment were ovariectomized females. One aim of future experimenters should be to determine if gonadal hormones and biological sex influence cells of the pVTA-AcbSh circuit, as suggested by the current literature [30–34] and the degree to which cells these cells contribute to novelty-mediated responses in each sex.

Together, these results suggest that cells of the pVTA are involved in novelty responses. However, it is still unclear how GABAergic and glutamatergic cells of the pVTA influence dopaminergic actions in response to novel stimuli. Future experiments should additionally aim to replicate and expand on the current results by testing rodents under other behavioral paradigms related to reward or depressive-like behaviors and including electrophysiological methods to provide further insight into the functional contributions of the VTA-AcbSh circuit generally as well as the contributions of specific pVTA subpopulations.

## Conclusions

The current report demonstrates that GFP/Cre recombinase from the AcbSh retrograde transports to the VTA region, and correctly orients hM3D(Gq)-mCherry to allow the selective expression in the pVTA. Stimulation of hM3D(Gq) receptors within the pVTA-AcbSh circuitry, prolonged locomotor responses to novelty without altering locomotor activity levels or auditory processing. These results add to the current literature defining the antero-posterior heterogeneity of the VTA and specifically, the role of the pVTA in establishing responses to novelty.
